# Adolescents Environmental Emotion Perception by Integrating EEG and Eye Movements

**DOI:** 10.3389/fnbot.2019.00046

**Published:** 2019-06-26

**Authors:** Yuanyuan Su, Wenchao Li, Ning Bi, Zhao Lv

**Affiliations:** ^1^Department of Design, Anhui University, Hefei, China; ^2^College of Design, Iowa State University, Ames, IA, United States; ^3^School of Computer Science and Technology, Anhui University, Hefei, China; ^4^School of Computer Science, Georgia Institute of Technology, Atlanta, GA, United States; ^5^Institute of Physical Science and Information Technology, Anhui University, Hefei, China

**Keywords:** electroencephalograph (EEG), eye movements, human-robot interaction (HRI), adolescents, environmental emotion perception

## Abstract

Giving a robot the ability to perceive emotion in its environment can improve human-robot interaction (HRI), thereby facilitating more human-like communication. To achieve emotion recognition in different built environments for adolescents, we propose a multi-modal emotion intensity perception method using an integration of electroencephalography (EEG) and eye movement information. Specifically, we first develop a new stimulus video selection method based on computation of normalized arousal and valence scores according to subjective feedback from participants. Then, we establish a valence perception sub-model and an arousal sub-model by collecting and analyzing emotional EEG and eye movement signals, respectively. We employ this dual recognition method to perceive emotional intensities synchronously in two dimensions. In the laboratory environment, the best recognition accuracies of the modality fusion for the arousal and valence dimensions are 72.8 and 69.3%. The experimental results validate the feasibility of the proposed multi-modal emotion recognition method for environment emotion intensity perception. This promising tool not only achieves more accurate emotion perception for HRI systems but also provides an alternative approach to quantitatively assess environmental psychology.

## 1. Introduction

Human-robot interaction (HRI) refers to the process of communication between a user and a robot, in which the user interacts with the robot via a certain “dialogue” language (Sloman, 2009; Nishiguchi et al., 2017; Khan et al., 2018). To date, HRI methods have been widely applied to the design of service robots, amusement robots, healthcare robots, and so on (Royakkers and van Est, 2015; Li et al., 2018; Zhang et al., 2019). Those robots are able to perfectly execute interactions with the user; however, they have a limited ability to adjust the interaction mode in response to the user's psychological states owing to their weak emotion perception (Pessoa, 2017; Cavallo et al., 2018). Effective recognition of users' emotional states will be important for improving the performance of HRI (Breazeal, 2003; Schaaff and Schultz, 2009; Boucenna et al., 2014a,b). For instance, if a healthcare robot could accurately perceive the user's emotional state, it could perform the corresponding feedback to enhance rehabilitation effectiveness. Therefore, establishing an emotional HRI method, especially for the social environment, has become a research hotspot in the field of robotics.

To achieve this purpose, bioinformation-based emotion perception and evaluation have received increasing attention (Picard et al., 2001; Kim and André, 2006, 2008; Wackermann et al., 2014; Peng and Lu, 2017). Generally, the acquisition of emotion-related bioinformation can be categorized into contact-free and contact methods. Contact-free methods refer to the recognition based on speech, facial expressions, human postures, etc. This type of method has the advantages of simple signal acquisition and a high comfort level for participants. However, when the participants intend to hide their emotions, contact-free methods cannot make a correct adjustment because the real emotion is not consistent with the external performance. By contrast, contact methods can effectively overcome the above-mentioned problem owing to the undeceptiveness of physiological signals. Currently, the main peripheral physiological signals acquired from contact bio-electrodes are electrocardiogram (ECG), electrooculography (EOG), electromyography (EMG), skin conductance (SC), and respiration. This bio-information has been applied to evaluate psychological effects, restorative results, and satisfaction degree in different environments (Hartig et al., 2003; Tyrväinen et al., 2014; Wang et al., 2016).

Electroencephalography (EEG) signals generated from the brain in the central nervous system have shown better performance in emotional HRI than peripheral bio-signals (Lin et al., 2010; Ravindra and Castellini, 2014; Wang et al., 2014). Besides, as a non-invasive measurement, EEG has become a valuable research tool for landscape architects in environmental science (Le Van Quyen, 2011; Righi et al., 2017). At present, EEG-based emotion perception approaches can be classified into two categories. One approach is to analyze emotion-related brain activities by observing the raw signal output, especially alpha wave and prefrontal asymmetry. For instance, Ulrich et al. compared the physical and psychological states of two groups living in a built natural environment and a built business/industry environment, respectively. They showed that the sample group in the natural environment had a higher alpha ratio than the other group, by integrating the results of questionnaires, interviews, brain activities, heart rate, and blood pressure (Ulrich et al., 1991). Nakamura and Fujii analyzed the quantity of alpha and beta rhythms induced by two different types of fence, i.e., consisting of closely growing bushes and concrete blocks, respectively. They concluded that the alpha ratio in the former case was higher than the latter (Nakamura and Fujii, 1992). Another approach is to obtain emotional states by feature extraction and classification of EEG signals collected in different environments. For example, Aspinall et al. used the commercial equipment Emotive to record five-channel emotion EEG signals in two dimensions while the participants were walking in a commercial street and a green space, respectively (Aspinall et al., 2015). Roe et al. (2013) confirmed that green space resulted in lower arousal and higher positive preference than an urban scene.

The aforementioned emotion perception methods mainly concern the recognition of discrete emotions (such as sadness, joy, depression, anger, etc.). However, in the field of environmental psychology, it is difficult to use these discrete emotional states to describe emotional activities sufficiently, owing to their complexity. Besides, such emotion recognition methods focus on exploration of the relationship between a general group and a specific environment (e.g., green space or commercial region), while ignoring the relationship between the general environment and a specific group (e.g., adolescents or the elderly). In fact, the latter has a more important role than the former in the design of HRI with “social intelligence.” Owing to the inseparable relationship between EEG and human emotions, EEG-based emotion recognition has attracted the attention of many researchers. In recent years, a large number of studies have demonstrated its superiority and stability (Nie et al., 2011; Jirayucharoensak et al., 2014; Zheng et al., unpublished). Studies have also shown that eye movement characteristics (i.e., blink frequency, saccade duration, pupil diameter, etc.) can reflect current emotional state to a great extent, and provide an efficient approach to recording users' behaviors (Lu et al., 2015). Considering the fact that adolescents, as a special group, may be sensitive to particular environments, we therefore selected different living environments to which they are often exposed as research objects. On this basis, we propose a new multi-modal environment emotion intensity perception method using information from EEG and eye movements for implementation of an emotional HRI. Specifically, we first design a stimulus selection method to induce different emotional states. Subsequently, we non-linearly scale the emotion intensity into five levels based on responses to different stimulus videos. Furthermore, we develop a dual method comprising a valence perception sub-model and an arousal sub-model to synchronously perceive emotion intensities in both two dimensions.

This paper is organized as follows. Section 2 introduces the generation of EEG signals as well as the emotion classification method. Section 3 depicts the procedure of data preparation, including the stimulus selection method and the experimental paradigm design. Section 4 details the proposed emotion intensity perception method using EEG and eye movement signals. The experiments are described in section 5 and section 6 concludes the paper.

## 2. Preliminaries

### 2.1. EEG Generation

**Figure 1:**
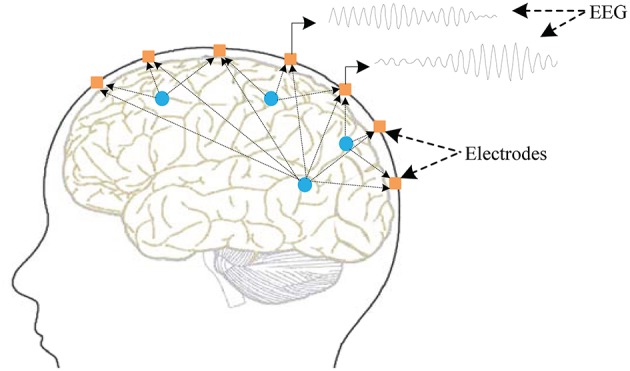
Fundamental principles of EEG generation. The potential presenting on the surface of the cerebral hemispheres is generated by the summed activity of hundreds of nerve cells. The bio-electrodes (black points) placed on the cerebral cortex are used to collect these potentials.

The brain consists of the cerebellum and the brain stem. The two hemispheres are separated by the longitudinal crack and connected by a large fibrous band named the corpus callosum in the middle. The surfaces of the brain are particularly complex and separated into pieces by a number of fissures, the largest of which are the Rolandic and Sylvian. The complex fissures make the surface area of the brain more than twice the size of a smooth one, and so are the number of neurons. A gray structure composed of nerve cells covers the surface of the cerebral hemisphere and forms the cerebral cortex, which is the center of the advanced nervous system. Subcortical nerve fibers access the brain and other parts of the body. Parts of the cortex are involved in specific functions, for example, visual information is dealt with by the occipital area, and auditory information is processed by the temporal lobe. For instance, the potential generated by the summed activity of hundreds or thousands of nerve cells will present on the surface of the cerebral hemispheres when the individual is viewing scenes or listening to sounds. Furthermore, this spontaneous potential can be collected by bio-electrodes placed on the cortex and recorded as EEG signals (Cooper et al., 2014). Figure 1 illustrates the generation principles of EEG.

### 2.2. Emotion Classification

**Figure 2:**
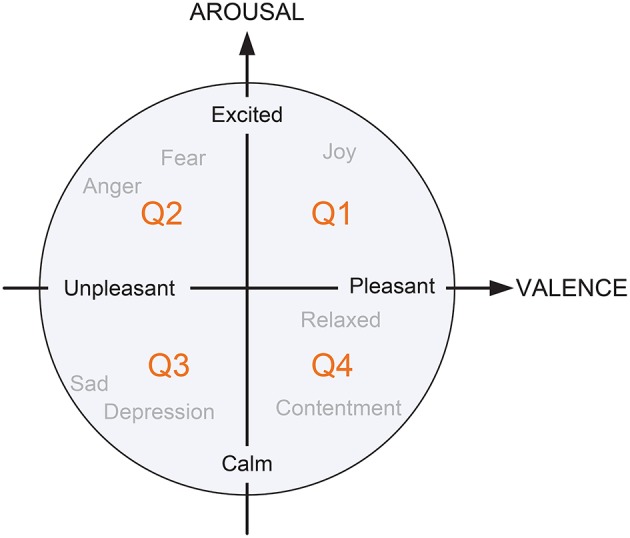
A simplified 2-D emotion model based on Russell's theory with horizontal dimension “VALENCE” and vertical dimension “AROUSAL.”

With the development of emotional generation and expression theories, various emotion classification models have been presented in the field of affective computing (Picard, 2000). Inspired by the Darwinian theory, Ekman defined six basic emotional states: happiness, surprise, anger, disgust, sadness, and fear. Generally, an individual's emotions are a mixture of two or more discrete representations. For instance, disappointment can be expressed as a combination of surprise and sadness (Ekman et al., 1987). However, there is a limitation to this approach, in that Ekman's definition cannot exactly describe complex emotional states. To address this issue, psychologists have employed *n*-dimensional spaces to represent emotion. For instance, a two-dimensional (2-D) valence/arousal space model (see Figure 2) is widely adopted in environmental psychology analysis (Russell, 1979; Russell and Snodgrass, 1987). Specifically, the “VALENCE” axis is associated with a negative or positive situation, which ranges from unpleasant to pleasant; the “AROUSAL” axis is related to the intensity of excitement (from calm to excited) according to the sensory stimulation. In this way, emotion categories, such as joy, fear, anger, sad, depression, contentment, and relaxation can be expressed in four quadrants in response to the degree and intensity of the two dimensions. Considering the complexity of emotional states induced by different built environments, we used a 2-D model to describe emotion intensity in the current research.

## 3. Data Preparation

### 3.1. Stimuli Selection

During the selection of stimulus videos, we are concerned with an HRI aiming at adolescents' living environments. The contents of the video database include both indoor space (e.g., bookstores, classrooms, libraries, shopping malls, KTV, bars, restaurants) and outdoor environments (e.g., commercial streets, urban roads, green spaces, playgrounds, garbage). We also present the everyday state of each scene as much as possible, not avoiding the occurrence of people and sounds. For example, the green space near a lake has a breeze, the library is relatively static, commercial street is mussy, etc. A total of 180 original video clips were used, and the duration was about 35 min. One hundred and twenty one different living scenes were recorded; the rest were collected from movies. In order to ensure effective elicitation of different emotional states, the stimuli used in our experiments were selected by the following steps.

First, to avoid different emotional states being induced in the same video segment, we kept the clips as short as possible. Thus, 55-s highlighted video segments with maximum emotional content from each of the original stimuli were manually extracted. Then, we selected 120 test clips preliminarily from 180 highlighted videos using self-assessment software designed in our laboratory. Employing this software, the user can control the mouse to click on different checkboxes to express the intensity of their emotional response after viewing a stimulus video. Furthermore, to determine the emotional intensity in response to the different built environments, we scaled the emotions into five levels based on the degree of arousal and valence dimensions, as follows.

- Arousal dimension: low degree arousal (level 1), low-medium degree arousal (level 2), medium degree arousal (level 3), medium-high degree arousal (level 4), high degree arousal (level 5);
- Valence dimension: low degree valence (level 1), low-medium degree valence (level 2), medium degree valence (level 3), medium-high degree valence (level 4), and high degree valence (level 5).

**Figure 3:**
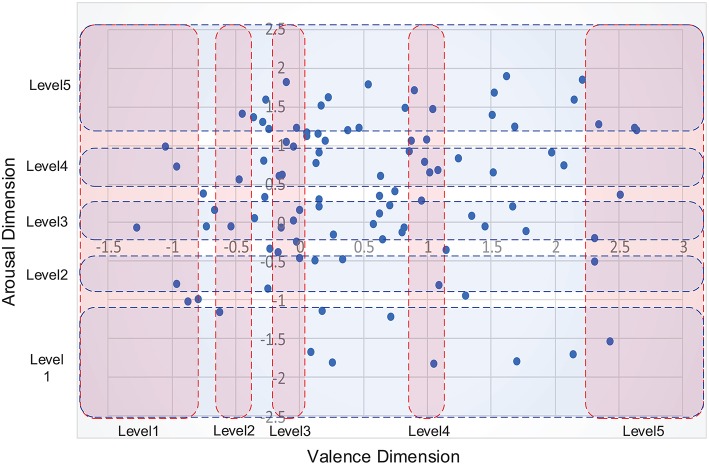
Illustration of stimulus selection method using a normalized 2-D valence/arousal space model. The x- and y-axis indicate the score values computed using Equation (1) according to the valence and arousal dimensions, respectively. Each blue dot corresponds to a stimulus video. The red and blue rectangles represent five predefined scopes for different emotion intensities in the arousal and valence dimensions, respectively.

On this basis, we further chose the 40 most representative stimulus videos from the 120 optimized test clips to improve the effectiveness of the stimulus video. Specifically, we first displayed these stimuli videos at random and asked each subject to view them as many times as he/she desired. Next, we computed the normalized arousal *score*_*a*_(*x*) and valence *score*_*v*_(*x*) for each stimulus video using the following equation:

(1) $$\begin{array}{r} score_{a}\left(x\right)=\frac{\mu_{ax}}{\sigma_{ax}},score_{v}\left(x\right)=\frac{\mu_{vx}}{\sigma_{vx}} \end{array}$$

where μ_*ax*_ and μ_*vx*_ denote the mean rating, and σ_*ax*_ and σ_*vx*_ indicate the standard deviation. The stimulus selection method is illustrated in Figure 3.

**Figure 4:**
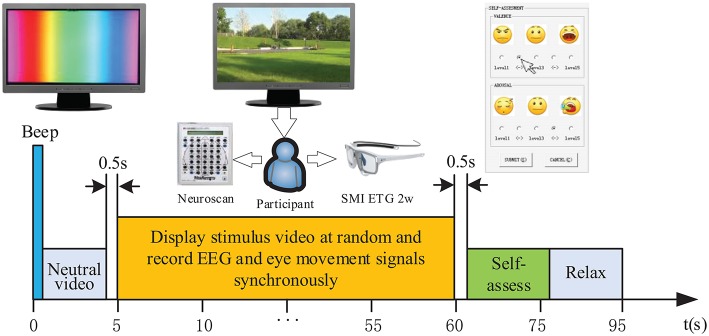
Demonstration of a single experimental paradigm.

In the proposed emotion perception method, we non-linearly scaled the emotions into five levels according to the arousal and valence intensities. As can be seen in Figure 4, some extreme emotional states were seldom induced because the story plots were not included in the visual built environments. As a result, fewer stimulus videos were located in the areas of low valence and high arousal (the corresponding emotion is “Anger,” as shown in Figure 2) compared with other areas. In accordance with this distribution, we selected five representative areas on each dimension of the 2-D model and determined eight optimum sample points as training data in each area, as follows.

- On the arousal dimension: eight training samples that were closest to the origin on the horizontal axis were chosen as level 3 (medium) stimuli, videos that were closest to ±2.5 on the horizontal axis were chosen as level 1 (low) and level 5 (high) stimuli, and videos that were closest to ±0.7 on the horizontal axis were chosen as level 2 (low-medium) and level 4 (medium-high) stimuli, respectively;
- On the valence dimension: eight training samples that were closest to the origin on the vertical axis were chosen as level 3 (medium) stimuli, videos that were closest to −1.5 and 3 on the vertical axis were chosen as the level 1 (low) and level 5 (high) stimuli, and videos that were closest to −0.4 and 1 on the vertical axis were chosen as level 2 (low-medium) and level 4 (medium-high) stimuli, respectively.

Compared with the arousal dimension, the divisions on the valence dimension were asymmetric because the stimulus distribution tended toward higher emotion intensity.

### 3.2. Experimental Paradigm Design

Before the experiment, all participants were informed about the purpose of this experiment and told the meanings of the terms “valence” and “arousal.” Then, they were trained to be familiar with the self-assessment software and the interaction method. To ensure the effectiveness of the induced emotion, the bias caused by different emotional states was suppressed by displaying a neutral video showing colored bands between different stimuli. Specifically, each trial started with a 5-s neutral video, followed by a 20 ms warning tone (“beep”). After 0.5 s, the videos were displayed at random and EEG and eye movement signals were recorded synchronously. During this step, the experiment could be paused at any time if the subject was made uncomfortable by the video content, experimental conditions, etc. When the participant finished viewing the stimulus, they were asked to evaluate their emotional intensity using the self-assessment software. After completing the above steps, the participant was allowed to relax to improve their performance on the next trial. The duration of a single experimental paradigm was ~95 s. This duration includes the time required to show the neutral video, to play the emotional clips, to perform the self-assessment, and to have a break. Considering the availability of emotional data, generally, the ideal total experimental time is <30 min in order to avoid physical or physiological fatigue. A single experimental paradigm is shown in Figure 4.

### 3.3. Data Acquisition

The EEG data used in our study were collected from healthy participants. Most of whom were undergraduate or graduate students from the College of Arts or College of Computer Science at Anhui University. A 30-channel EEG acquisition instrument (NeuroScan, Inc., El Paso, TX) was used, and the distribution of electrodes was in line with the international 10–20 system (Homan et al., 1987). The reference electrodes were placed on the right and left mastoids, respectively, and the ground electrode was arranged on the forehead. In the aspect of eye movements acquisition, we used the SMI ETG 2w eye-tracking glasses to record the participant's natural gaze behaviors due to its high robustness, mobility, and ease of use. Besides, it could also automatically compute eye movement features, such as saccade, fixation, and blink, which provided a convenience for eye movement-based emotional features extraction. It was worth noting that a specific trigging software developed in our laboratory was used to start two acquisition devices in order to record EEG and video eye movement signals synchronously.

## 4. Methods

**Figure 5:**
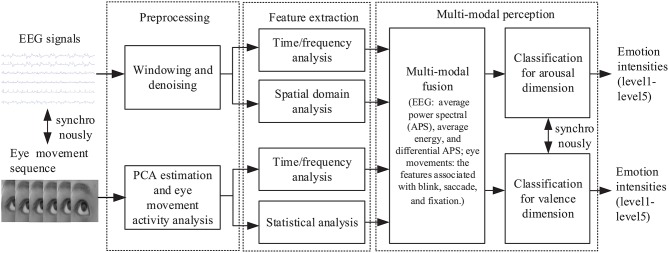
Overall architecture of the proposed environment emotion perception method for HRI using a combination of EEG and eye movements.

First, we synchronously collected the EEG and eye movement signals induced by different stimuli. Then, we pre-processed two modal signals and extracted their feature parameters using time/frequency or spatial domain analysis. On this basis, we established the valence and arousal perception sub-models, respectively. In the recognition stage, we used this dual sub-model to classify emotional intensities synchronously in two dimensions, ranging from level 1 to 5. The overall architecture of the proposed method is shown in Figure 5.

### 4.1. Preprocessing

#### 4.1.1. Frame Blocking and Windowing for EEG Signals

To ensure the short-term stationarity of the observation EEG signals, frame blocking and sliding window techniques were used. Suppose that *x*(*n*) is a finite duration segment of emotional EEG signals. Then the basic windowing procedure can be depicted as:

(2) $$\begin{array}{r} \hat{x}\left(n\right)=x\left(n\right)\omega \left(n\right) \end{array}$$

where $\hat{x}\left(n\right)$ denotes the windowed signals and ω(*n*) denotes a window function. To reduce signal discontinuities between each independent frame as much as possible, we adopted the Hamming window as the window function, defined as follows Lv et al. (2010):

(3) $$\omega (n)=\{\begin{array}{c} 0.54-0.46\cos[2\pi n/\left(N-1\right)],\;0\leq n\leq N-1\\ 0,\;otherwise \end{array}$$

**Figure 6:**
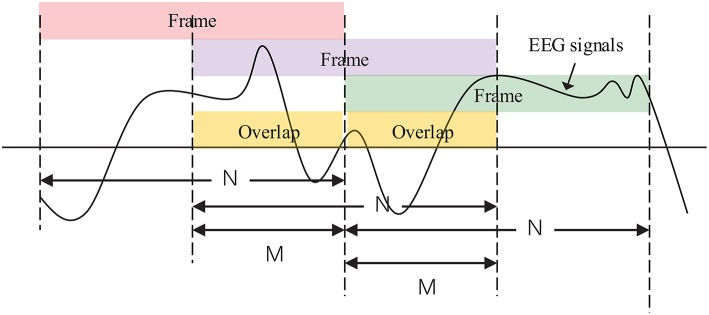
EEG signals are windowed into frames of *N* samples and overlaps of *M* samples in the case of *M* = (1/2)*N*.

Considering the balance between computation load and online performance, the number of overlapping samples was empirically initialized to half the length of the frame. The basic windowing procedure is illustrated in Figure 6.

#### 4.1.2. Denoising

Emotional EEG and eye movement signals are susceptible to interference from various sources of noises during data collection. In order to preserve the effective emotional component and suppress the influence of noise, we performed a denoising operation. Specifically, for the EEG signals, we manually removed any trials seriously affected by artifacts and used a 32-order linear phase finite impulse response filter with cut-off frequency of 0.5–60 Hz to process the windowed signals. As the effects of illumination reflection on the diameter of the pupil can influence emotional information, we also used principal component analysis to estimate the illumination reflection of the pupil diameter and remove this component based on the original eye movement data (Soleymani et al., 2012).

### 4.2. Feature Extraction

#### 4.2.1. Time-Frequency Domain Features

**Figure 7:**
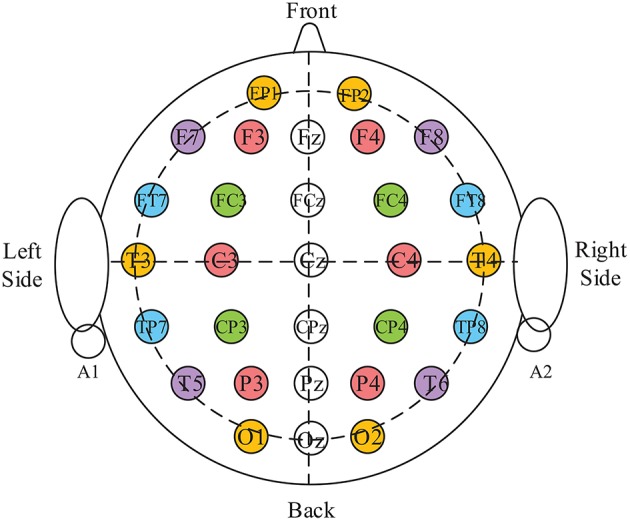
Demonstration of symmetrical pairs of collection electrodes. The same color located on the horizontal direction indicates a pair of electrodes; circles with numbers represent the electrodes' names and locations.

EEG signals can be divided into six independent frequency bands on the basis of the functions of the bands, i.e., delta (0.5–4 Hz), theta (4–8 Hz), alpha (8–12 Hz), slow alpha (8–10 Hz), beta (12–30Hz), and gamma (30–40 Hz). As these frequency bands have been shown to be associated with the variability of emotion (Ulrich et al., 1991; Droit-Volet, 2013), we extracted the average power spectral (APS) and average energy (AE) from the theta, alpha, beta, and gamma frequency bands, respectively, as feature parameters. Given that the possible asymmetry of parameters between the left and right hemispheres is closely related to the degree of valence, the differential average spectral power (DAPS, from all frequency bands other than slow alpha wave) values between all symmetrical pairs of electrodes were extracted as supplementary feature parameters. The distribution of symmetrical pairs of electrodes is shown in Figure 7. Consequently, we acquired 420 dimensions of (30*6+30*6+12*5) EEG features from an individual frame in the case of a 30-electrode arrangement.

To extract emotional features from the eye movement information, we first applied a short-term Fourier transform to obtain the power spectral density, as well as the differential entropy of the pupil diameter data in terms of four frequency bands (0.6–1.0, 0.4–0.6, 0.2–0.4, and 0.01–0.2 Hz) (Soleymani et al., 2012). We also computed some conventional time-domain features, such as the standard deviation and mean of duration for three basic eye movement activities (i.e., saccade, blink, and fixation). In order to obtain more potential emotional information, the detailed event statistics were considered as features. In this way, we acquired a total of 20 dimensions of features from eye movement data. The details of the features extracted from EEG and eye movements are listed in Table 1.

**Table 1:** Descriptions of extracted time-frequency domain emotional features.

**Features**	**Dimensions**	**Descriptions**
EEG: Average power spectral (APS)	30^*^6	32: Number of bio-electrodes. 6: APS from delta, theta, slow alpha, alpha, beta, and gamma frequency bands, respectively.
EEG: Average energy (AE)	30^*^6	32: Number of bio-electrodes. 6: AE from delta, theta, slow alpha, alpha, beta, and gamma frequency bands, respectively.
EEG: Differential APS (DAPS)	12^*^5	12: Number of symmetrical pairs of bio-electrodes, i.e., FP1-FP2, F7-F8, F3-F4, FT7-FT8, FC3-FC4, T3-T4, C3-C4, TP7-TP8, CP3-CP4, T5-T6, P3-P4, O1-O2. 5: DAPS from all frequency bands except slow alpha wave.
Eye movement: Features of blink	1^*^2	2: Mean and deviation of duration, respectively.
Eye movement: Features of saccade	1^*^3	3: Mean and deviation of duration, and rotation angle, respectively.
Eye movement: Features of fixation	1^*^2	2: Mean and deviation of duration, respectively.
Eye movement: Statistical features	1^*^13	13: Frequency of saccade, blink, and fixation; duration maximum, duration average, duration minimum, rotation angle maximum, and rotation angle average of fixation and saccade.

#### 4.2.2. Spatial Domain Features

We performed independent component analysis (ICA) on a single trial **x**_*n*_ (*n*=1,…,*N*), where *N* is the number of EEG trials. The information maximization (Infomax) criterion and natural gradient algorithm were applied to optimize the unmixing matrix **W** (Wu et al., 2018). To acquire the maximum projected position of ICs, we calculated the absolute value of the mixing matrix **A**_*n*_ (${\text{A}}_{n}={\text{W}}_{n}^{-1}$) and saved the index of maximum values into a matrix **D**_1 × 30_ according to each column vector $\left|{\text{a}}_{h}^{n}\right|$(*h*=1,2,…,30) of |**A**_*n*_|. In particular, if the matrix **D**_1 × 30_ included the index of the whole channel simultaneously, we inferred that the spatial filter was valid. For a valid filter **W**_*n*_ with conformed validity judgment, we chose the corresponding columns in the filter according to the values of **D**_1 × 30_. In this way, the ICA filter bank $\left\{W_{1}^{n},...,W_{30}^{n}\right\}$ was established. We employed a mean filter (the mean value of all valid filters) to linearly project each trial and extract ICs $\left(u_{1}^{n},...,u_{30}^{n}\right)$ for different emotional states. Letting $\hat{S}=[u_{1}^{n},...,u_{30}^{n}]$, we performed singular value decomposition on $\hat{S}$, with **ES** regarded as the spatial features.

(4) $$\{\begin{array}{l} \hat{S}=\text{U}\Sigma {\text{V}}^{T}\\ ES=[\lambda_{1}v_{1},...,\lambda_{30}v_{30}] \end{array}$$

where **U**, **V** are orthogonal matrices, **Σ** is a diagonal matrix, λ is the diagonal element of **Σ**, and **v** is a column vector of **V**.

Furthermore, in order to reduce differences among different participants, all feature parameters were normalized within the range of [0, 1]. That is, we first computed the absolute values of all feature vectors from each participant, then subtracted the minimum and maximum and calculated the difference values; finally, each feature vector was divided by this difference value to give the normalized results.

### 4.3. Multi-Modal Fusion and Classification Strategy

In the proposed method, we performed a two-classification scheme to perceive emotion intensity based on a support vector machine (SVM). One classification was based on arousal recognition for five levels: calmness (level 1) to excitement (level 5). The other classification was based on valence recognition, ranging from unpleasantness (level 1) to pleasantness (level 5). In the training phase, all of the training data were used to classify the stimuli on the arousal and valence dimensions for levels 1–5. In the recognition phase, when the new observation sample was input, the trained SVM methods were used to classify emotional intensity based on the arousal and valence dimensions synchronously. In order to combine the EEG and eye movement information, we applied two classifiers, for EEG and eye movement signals, respectively. A decision-level fusion [DLF; i.e., fuzzy fusion strategy (Murofushi and Sugeno, 1989)] was used to integrate them in order to achieve high recognition accuracy. The detailed procedure was as follows.

**Definition 1**. A fuzzy measure *μ* defined on a finite index set *X* = {*x*_1_, *x*_2_, …, *x*_*n*_} is a set function *μ*:*G*(*X*) → [0,1](*G*(*X*) is the power set of *X*) satisfying:

(5) $$\{\begin{array}{c} \mu (\emptyset )=0,\mu (X)=1,\\ A\subseteq B\Rightarrow \mu (A)\leq \mu (B) \end{array}$$

In the present work, we use the discrete Choquet integral proposed by Murofushi (Murofushi and Sugeno, 1989).

**Definition 2**. Assume that μ is a fuzzy element on the index set *X*. The discrete Choquet integral using the function *f* : *X* → ℝ^+^ regarding the variable μ can be defined as follows:

(6) $$\begin{array}{r} \zeta_{\mu }\left(f\left(x_{1}\right),f\left(x_{2}\right),\ldots ,f\left(x_{n}\right)\right):=\overset{n}{\underset{i=1}{\sum }}\left[f\left(x_{\left(i\right)}\right)-f\left(x_{\left(i-1\right)}\right)\right]\mu \left(A_{\left(i\right)}\right) \end{array}$$

Here, ·(*i*) indicates the index after permutation, which should satisfy the following relationship:

(7) $$\begin{array}{r} 0\leq f\left(x_{\left(1\right)}\right)\leq f\left(x_{\left(2\right)}\right)\leq \cdot \cdot \cdot \leq f\left(x_{\left(n\right)}\right)\leq 1 \end{array}$$

and

(8) $$\begin{array}{r} f\left(x_{\left(0\right)}\right)=0andA_{\left(i\right)}:=\left\{x_{\left(i\right)},x_{\left(i+1\right)},\ldots ,x_{\left(n\right)}\right\} \end{array}$$

Suppose that *C*_1_, *C*_2_, …, *C*_*m*_, belong to *m* classes and $X^{T}=\left[x_{1}\ldots x_{n}\right]$ are composed of *n*-dimensional vectors. A confidence factor will be obtained by the function $\Phi_{i}^{j}\left(X^{\circ }\right)$ for each classifier. In order to combine all confidence factors, we further define a global confidence factor as follows:

(9) $$\begin{array}{r} \Phi_{\mu_{j}}\left(C_{j};X^{\circ }\right):=\zeta_{\mu_{j}}\left(\Phi_{1}^{j},\Phi_{2}^{j},\ldots ,\Phi_{n}^{j}\right) \end{array}$$

where *C*_*j*_” and *u*^*j*^(*j* ∈ {1, 2, …, *m*}) indicate the class and its importance, respectively. Finally, the class of the unknown sample *X*° can be obtained by its correspondence to the highest confidence factor.

The core problem is to acquire the fuzzy element μ with *m*(2^*n*^ − 2) coefficients. For clarification, we set the number of classes to be 2 and compute μ by defining the minimizing error function, that is,

(10) $$\begin{array}{r} J=\overset{l_{1}}{\underset{k=1}{\sum }}{\left(\Phi_{\mu^{1}}\left(C_{1};X_{k}^{1}\right)-\Phi_{\mu^{1}}\left(C_{2};X_{k}^{1}\right)-1\right)}^{2}\\ +\overset{l_{2}}{\underset{k=1}{\sum }}{\left(\Phi_{\mu^{2}}\left(C_{2};X_{k}^{2}\right)-\Phi_{\mu^{1}}\left(C_{1};X_{k}^{2}\right)-1\right)}^{2} \end{array}$$

where *l* = *l*_1_ + *l*_2_ are training examples and their labels are $X_{1}^{j},X_{2}^{j},\ldots ,X_{l_{j}}^{j},j=1,2.$, respectively. We rewrite this in terms of a quadratic optimization problem in the case of 2*(2^*n*^ − 2) variables and 2n*(2^*n*−1^ − 1) constraints:

(11) $$\begin{array}{r} \begin{array}{c} \;minimize\frac{1}{2}\mu^{T}D\mu +\Gamma^{T}\mu \\ under\;the\;constraint\;A\mu +b\geq 0 \end{array} \end{array}$$

where μ is a vector with 2*(2^*n*^ − 2) dimensions, which involves all of the fuzzy elements μ^1^,μ^2^,i.e., $\mu :=\left[\mu_{1}^{T}\mu_{2}^{T}\right]$, with

(12) $$\begin{array}{l} \;\mu^{j}:=[\mu^{j}(\{x_{1}\})\mu^{j}(\{x_{2}\})\ldots \mu^{j}(\{x_{n}\})\\ \mu^{j}(\{x_{1},x_{2}\})\ldots \mu^{j}(\{x_{n-1},x_{n}\})\ldots \mu^{j}(\{x_{2},x_{3},\ldots ,x_{n}\}){]}^{T} \end{array}$$

Consequently, we can obtain the optimum fuzzy elements representing the level of importance for each classifier.

## 5. Experiments and Results

Twelve participants (seven male and five female) aged between 17 and 26 years (mean = 22.3, *SD* = 4.1) were involved in our experiments. Forty emotional trials were performed for each participant. Experiments were carried out in an illumination-controlled laboratory environment. Two dedicated computers were employed: one (T6400 @ 2.00 GHz, 4 G RAM) was used to record EEG signals, the other (i7-5500U @2.4 GHz, 8 G RAM) was used to display the videos, perform self-assessment, and transmit synchronization signals directly to the signal collection computer. All selected stimuli videos were randomly displayed on a 14-inch screen with 1280 × 1024 resolution and a 60-Hz refresh rate. In order to further suppress the influence of ocular artifacts, the screen resolution was set to 800 × 600 and the image filled approximately two-thirds of the screen. Moreover, every participant was required to sit about 1.0 m in front of the screen. Stereo loudspeakers were located on the desktop and the sound volume was empirically set at a reasonable level. Before the experiment, all participants were required to preview 2–3 videos to become familiar with the device and procedure. We also asked the participants whether the experimental environment (e.g., the distance between subject and screen, volume, indoor temperature) was comfortable. If the environment was not comfortable, the settings were adjusted on the basis of the participant's feedback.

### 5.1. Analysis of Raw EEG Signals

We performed EEG trials with three typical scenes (i.e., shopping mall, green space, and dumpsite) to analyze the differences in brain activities in different built environments. The average values of APS, AE, and DAPS for all frequency bands are shown in Table 2.

**Table 2:** Averaged APS, AE, and DAPS.

**Scenes**	**Valence**			**Arousal**		
	**APS**	**AE**	**DAPS**	**APS**	**AE**	**DAPS**
Shopping mall	31.28	25.72	0.04	23.83	18.90	0.22
Green space	14.79	9.23	0.91	11.75	10.48	1.53
Dumpsite	7.23	20.63	3.25	9.68	16.61	3.09

As can be seen in Table 2, there were clear variations in brain activities in different built environments. For instance, the participants retained relatively high valence in the shopping mall scene, the average APS was 31.28, while DAPS was as low as 0.04. On the contrary, the degree of valence decreased when they viewed the videos of the dumpsite (APS = 7.23, DAPS = 3.25). Similarly, the highest arousal values were recorded for the shopping mall scene (APS = 23.83, DAPS = 0.22) and the lowest values for the dumpsite scene (APS = 9.68, DAPS = 3.09). In contrast to APS and DAPS, the minimum values of AE were observed for the green space, i.e., 9.23 and 10.48, according to the valence and arousal dimensions, respectively. Participants reported that they felt calm and relaxed when watching the green space scene. Therefore, the energy from neural sources would be reduced compared with the other two scenes owing to the intensity decrement of brain activities.

### 5.2. Emotion Intensities Recognition

**Figure 8:**
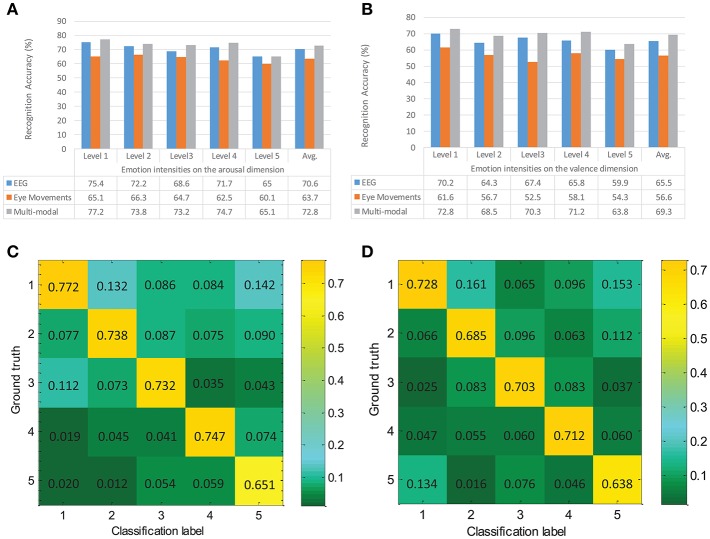
Emotion recognition results and normalized confusion matrix over all participants. Average accuracies in **(A)** the arousal dimension and **(B)** the valence dimension are presented, corresponding to EEG, eye movements, and multi-modal fusion (DLF), respectively. **(C,D)** show the confusion results for the arousal and valence dimensions. Numbers 1–5 indicate the emotion intensities corresponding to the predefined levels described in section 3.1.

In this experiment, the length of a frame was 500 sampling points (duration was 2 s) and the overlap was 250 points (duration was 1 s). The ground truth was directly obtained from the results of the self-assessment software. The linear radial basis function (RBF) kernel function was applied to the SVM method, and the penalty factor was empirically set to 1. The recognition results were compared with the data labels to acquire the classification accuracy ratio. If they were the same, the classification was considered to be correct; otherwise, it was wrong. A 10*5 cross-validation method was also employed to improve the reliability of the recognition results, as follows: all samples were sorted at random and divided into five parts, then one part was taken out as a test dataset and the remaining four parts were used to train the classifier; this procedure was repeated 10 times for each participants' data. The average emotional recognition results based on EEG, eye movements, and multi-modal fusion for different emotional intensities are shown in Figure 8.

In Figure 8A, the recognition accuracies in the arousal dimension are reported. The average emotion recognition accuracy based on EEG was 70.6%, which showed an increment of 6.9% compared with the eye movement result. For the performance of modality fusion, we achieved improvements of 2.2% and 9.1% for the EEG and eye movement information fusion. Similarly, in Figure 8B, 65.5 and 56.6% represent the average recognition ratios of EEG and eye movements on the valence dimension, respectively. The result obtained with the modality fusion method was 69.3%, higher than those for any single modality. It is clear that the modality fusion method showed superior performance to either the EEG or eye movement modality.

## 6. Discussion

The motivation of this work was to develop an environment emotion HRI method by establishing a multi-modal emotion perception algorithm combined with quantitative assessment of environmental psychology. To ensure the diversity of the stimuli, we considered the following factors in the design procedure: (1) recording the indoor and outdoor videos under different illuminations, weather conditions, and time periods in order to ensure diversity of stimuli; (2) avoiding manual intervention as far as possible when collecting stimulus videos so as to keep the scenes in a natural state; and (3) using stimulus videos without a storyline to ensure that the participant could focus effectively on the visual environments themselves. Thus, the distributions of emotional states presented unevenness in the 2-D emotion model space because the built environments could not induce extreme emotional responses, such as “anger” and “sadness” (located on the extremes of Q2 and Q3 in Figure 2, respectively). On the other hand, some types of intense emotions, such as “disgust” could be induced by certain environments, such as the dumpsite. The emotions induced by the built environments tended to be subtler and more complex compared with those induced by videos with specific content. In order to exactly describe the emotional variations, we established a dual emotion recognition model based on cognitive theory to perceive the intensities of arousal and valence in the current work.

The experimental results, shown in Table 2, revealed that the majority of adolescents presented higher intensity of brain activities (i.e., high-level valence and arousal) when watching the videos of shopping malls, sport games, or parties. That is, a higher level of excitement was induced on both the valence and arousal dimensions. Generally, short-term excitement can reduce inhibition via endocannabinoids (Pezzulo et al., 2013), which is beneficial to adolescents' health. However, long-term excitement may result in fatigue and even minor brain damage (Kaplan, 1995). In our experiment, most participants presented medium-low arousal and medium valence when viewing the landscape, an ideal and satisfied emotional state. This result is in line with the theory of green space as a restorative environment (Cohen-Cline et al., 2015).

The normalized confusion matrix over all participants is shown in Figures 8C,D. The values presented on the diagonal are correct recognition rates, and the off-diagonal denotes substitution errors. The largest between-class substitution errors were 0.142 and 0.161, while the smallest errors were 0.012 and 0.016 for the arousal and valence dimensions, respectively. By analyzing all trials, we found that most of these errors were falsely returned level 1 and 2 results.

Closer inspection of the confusion matrix revealed that the accuracy of the experiment was associated with the stimulus videos. As shown in Figure 8C, the between-class substitution errors for level 1, 2, and 3 were 0.112 (false return level 3), 0.132 (false return level 1), and 0.087 (false return level 2), respectively. In other words, the largest errors were all located within level 3. By analyzing the original stimulus videos, we observed that emotions of medium-low arousal were mainly induced by the calm-dominated videos (e.g., libraries, bookstores, green spaces). These kinds of environment have similar characteristics to medium-low arousal, that is, static space, quiet environment, single-component elements, neutral and uniform colors, etc. As a result, they are accepted by the participants as stimuli with medium-low arousal by consensus. Owing to differences in emotional tension and cognition, some unexpected classification results occurred within a reasonable range (especially for level 3). Furthermore, the lowest recognition ratio was located in the fifth column, while the largest between-class substitution error (0.142) was located in the first row. This result indicates that the probability of the highest arousal (excitement) being falsely reported as the lowest arousal (calm) was higher here than in other cases, which is consistent with the report published in 2012 (Soleymani et al., 2012) to some extent. Based on information regarding participants' emotional responses after viewing the stimuli, we speculate that discrepancies in adolescents' perceptual and rational choices may have had a major influence on our results.

In Figure 8D, the largest between-class substitution errors for level 1 and 5 are located on opposite sides: level 5 (0.153) and level 1 (0.134). This confusion may be caused by differences in the personalities and preferences of the adolescents. For instance, some young individuals may prefer the atmosphere of a music club or a bar, while others may be unwilling to stay in such environments. In contrast to those for levels 1 and 5, the recognition accuracy ratios are relatively stable for levels 2, 3, and 4. This reveals that the majority of adolescents have similar views of stimuli with medium-high valences, as most such stimulus videos depicted commercial complexes, playgrounds, green spaces, etc.

Generally, the participants achieve a higher consensus on the lowest level (level 1) for the arousal dimension, that is, they feel calm when staying in a peaceful environment. On the contrary, they present a bigger difference on the highest level (level 5) for the arousal dimension. We speculate that this result is associated with the cognition difference caused by character differences (e.g., introverted or extroverted) for the adolescent individual. In addition, different individuals may generate different cognitive responses to the same thing owing to different preferences and educational background. Therefore, the largest between-class substitution error on the valence dimension presented the opposite position, i.e., the right-top and left-bottom.

## 7. Conclusion

In the present work, we presented a multi-modal approach for synchronously perceiving emotional intensities in both the valence and arousal dimensions. On this basis, we further investigated the influence of different environments on adolescents' psychology. The experimental results indicated that the proposed method is a promising tool to implement an emotional HRI. Future work should prioritize improvements to perceive subtler emotions with less intervention for vulnerable populations. We also hope that it can be used as an auxiliary method to enhance the effectiveness of emotional restoration in different open levels, color levels, enclosing form of environmental types, etc. Using wearable EEG and eye movement equipment to detail emotional variation in healthy environments (such as therapeutic landscapes, healing gardens, meditative gardens, and restorative gardens) will be another important research direction in the future.

As the proposed multimodal-based HRI method can establish an effective emotional communication channel between human and robot, it has high potential for development of the following applications: (a) design of an intelligent e-healthcare robot to implement monitoring, diagnosis, and prevention of mental disorders; (b) development of an emotion safety evaluation robot for people engaged in high-risk work (e.g., drivers, pilots, soldiers); (c) implementation of a service robot for patients with motor diseases, such as amyotrophic lateral sclerosis or motor neuron disease, or injured vertebrae, who retain normal brain or eye movement responses.

## Ethics Statement

The research related to human use complied with all the relevant national regulations and institutional policies, was performed in accordance with the tenets of the Helsinki Declaration, and the experiments had received approval by the ethics committee of Anhui University.

## Informed Consent

Written informed consent was obtained from all individual participants included in the study.

## Author Contributions

YS, WL, and NB mainly focused on the development and verification of the proposed method. ZL collected, organized the literature, and supervised all the process. All authors read and approved the final manuscript.

### Conflict of Interest Statement

The authors declare that the research was conducted in the absence of any commercial or financial relationships that could be construed as a potential conflict of interest.
